# Neonatal Mastauxe (Breast Enlargement of the Newborn)

**Published:** 2013-07-01

**Authors:** V Raveenthiran

**Affiliations:** Department of Pediatric Surgery, SRM Medical College and Hospital SRM University, Chennai, India.

 Maternal estrogen is known to cause varying degree of breast enlargement in approximately 70% of newborn. [1] Usually the diameter of breast bud measures 1 to 2 cm in the first few weeks of life [2]. But Athena has seen some of these breasts of alarming size (Figure 1). The reason for this exaggerated response is unclear. These giant breasts will be hard and tender in contrast to the soft and painless gynecomastia of older children and adolescents. Postnatally, falling levels of maternal estrogen is thought to trigger prolactin secretion in the pituitary of the newborn [1,3]. Resultant prolactinemia stimulates neonatal breasts and causes milk secretion in 5 to 20% of newborn [4]. It is popularly known as witch’s milk because it is believed in folklore that witches and goblins would feed on it [5]. Witch’s milk resembles maternal milk with identical concentration of IgA, IgG, lactoferrin, lysozyme and lactalbumin [6]. Inadequate let-out of milk, either due to improper canalization of lactiferous ducts or due to lack of oxytocin stimulus in the newborn, may lead to stagnation of milk (galactocele). Superadded infection may result in complications such as mastitis and breast abscess [7]. Athena intended to update the recent developments on this oddity of the Nature. 

**Figure:**
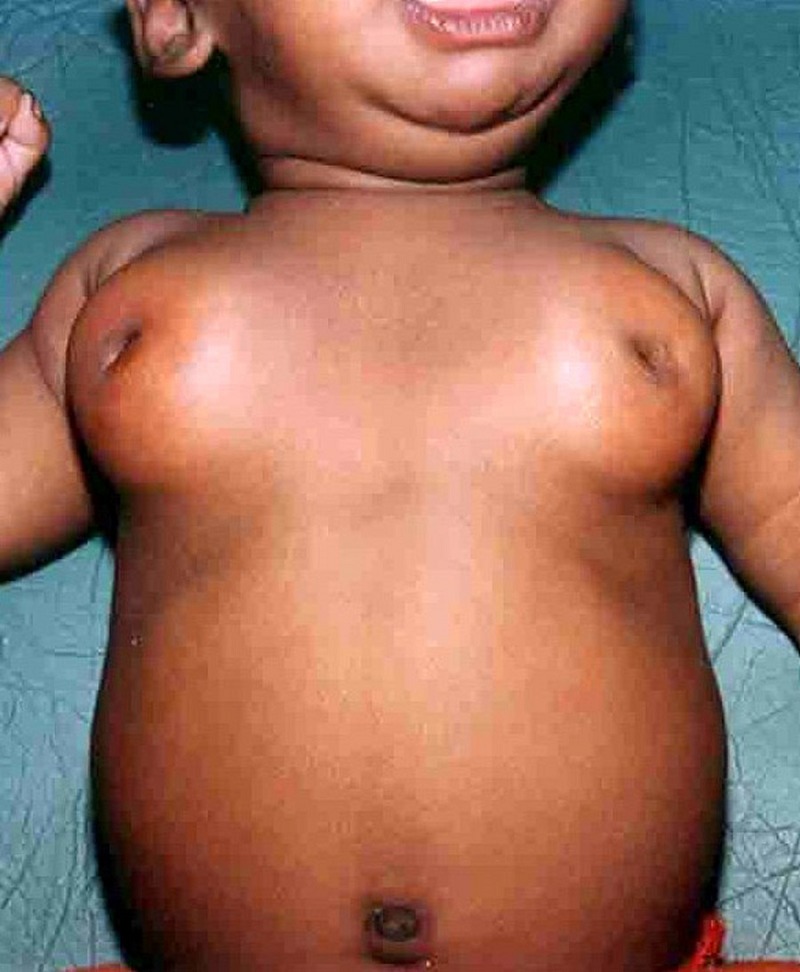
Figure 1: Giant Mastauxe of newborn

An extensive review of literature has left Athena much disappointed for many reasons. First of all, there is no proper terminology to describe uncomplicated, physiological enlargement of breasts in the newborn. Research papers seldom distinguish various forms of breast swellings in newborn such as physiological enlargement, exaggerated development, bulging galactocele, inflammatory swelling and breast abscess. Terms such as ‘mastitis’ [8,9,10], ‘galactorrhea’ [4,11], ‘gynecomastia’ [11,12], ‘galactocele’ [12,13], ‘breast hypertrophy’[14] and ‘breast enlargement’[1] have been used interchangeably to denote any of the aforementioned presentations. Notoriously, hardness (induration), hyperemia and tenderness of proliferating mammary glands are often mistaken for the signs of inflammation and hence the popular misnomer “Mastitis Neonatorum” [8]. Considerable overlap of the various presentations further mars the clarity. Imprecise terminology, lack of accurate definition and absence of diagnostic criteria have left much confusion in the literature [4]. Considerable etymological research prompted Athena to revive an obsolete term - “Mastauxe” (pronounced as mas-tawk′sē). It is a combination of two Greek words mastos (Breast) and auxein (increase in size) [15,16]. Semantically, it appears to be the right word to describe uncomplicated physiological breast enlargement of newborn under hormonal influence. The terms “neonatal mastitis”, “neonatal breast abscess” and “neonatal galactocele” are better reserved to denote complications of “neonatal mastauxe”. Exaggerated breast enlargement may be referred to as “giant mastauxe” (Table 1). 

**Figure:**
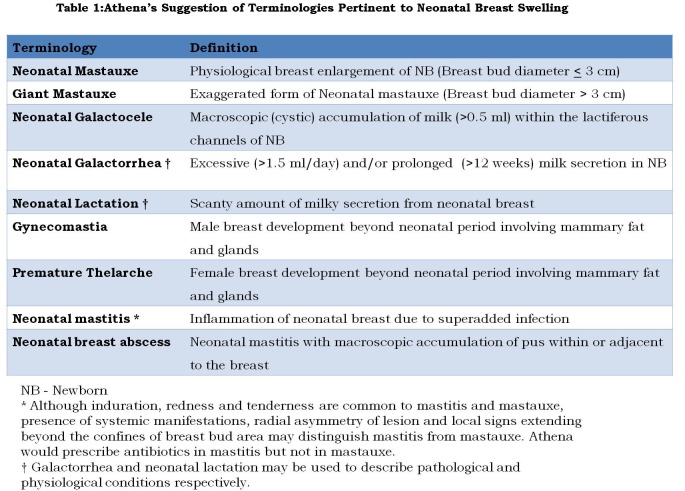
Table 1

Adding to the lexicological confusion, the literature is also undermined by poor quality research. Almost all the available papers are either anecdotal case reports or small case series. Large clinical trials and experimental studies are conspicuously missing over the last 5 decades. Three recently published case series [8,9,10] do not add anything significantly to the existing knowledge (Table 2). Commonality of the 3 papers can be summarized as follows: Neonatal mastitis is common in full term female neonates during the third and fourth week of life. Bilateral involvement is rare (< 10%). There is no side predilection. Maternal endocrinopathy was noted in 0 to 14% of cases. Staphylococcus is the commonest isolate accounting for more than 60% cases. With or without pretreatment, 50 to 70% of them progress to become breast abscess requiring needle aspiration or surgical drainage of pus. About 30 to 60% of the neonates needed hospital admission for intravenous antibiotics or for surgical treatment. Although systemic manifestations are generally rare (8 to 28%), serious life-threatening systemic complications such as cerebral abscess have been documented in the literature [17,18]. Ruwaili and Scolnik [9] noted that approximately one half of neonatal mastauxe is complicated by mastitis and one half of neonatal mastitis progresses to become frank abscess. Neonatal mastitis showed cluster occurrence in Brett’s series [8]. Long-term follow-up is uniformly missing in all the studies.

**Figure:**
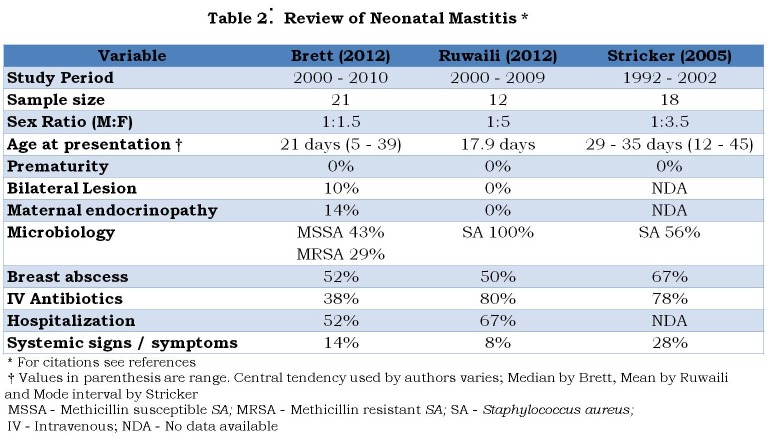
Table 2

It is often difficult to say with certainty as to when mastauxe becomes true mastitis because they frequently share the same physical signs. Athena is curious if modern imaging modalities can distinguish the two. Borders et al. [19] studied sonographic appearance of mastitis in 5 newborns. Breast buds were relatively hypoechoic in mastauxe while increased echogenicity is characteristic of mastitis. Breast abscesses may be either anechoic or echogenic depending upon the nature of their contents. Both abscess and mastitis show increased flow pattern of surrounding fat in color Doppler; but flow within the abscess is singularly absent. The authors recommended sonographic examination if the response to antibiotic is delayed or if there is a suspicion of abscess formation. Welch et al. [20] indicated that ovoid masses of anechoic areas with intervening septae could suggest duct ectasia especially when the septae show vascular flow in color Doppler. But these findings are based on a very small number of observations and they need to be reconfirmed by larger studies. Mastauxe is incidentally noted to exhibit increased uptake of Technetium99m pertechnetate during thyroid scintigraphy [21,22]. Practical significance of this interesting observation is yet to be studied. 

Mastitis or its surgical treatment may cause damage to developing breast bud. Impaired development and asymmetry of breasts in adulthood is traditionally considered to be a serious complication of neonatal mastitis [7]. Recently, Panteli et al. [23] provided a modest scientific evidence of this assertion. They followed-up 8 neonates with mastitis into their adolescence. Seven of them had undergone surgical drainage of neonatal breast abscess. The mean age at follow-up was 14 years (range 10 - 15 yrs). Half of the patients had abnormal finding in clinical and/ or sonographic examination. This included reduced breast size in 2 (25%), altered breast texture in 4 (50%) and breast asymmetry in 1 (13%). This paper underlines the importance of prompt intervention and long-term follow-up of neonatal mastitis.

The mystery as to why some neonatal breasts show exaggerated response to hormones is unclear. It is probably attributable to hypersensitivity of breast tissue to estrogen and/ or prolactin. If this hypothesis is true, estrogen being a known carcinogen, giant mastauxe will be more vulnerable for malignant change in adulthood. French molecular oncologists have shed some light on this important question. Recently they showed that both BRCA1 and BRCA2 (tumor suppressor genes of breast cancer) are over expressed in infantile gynecomastia of a 2-year-old boy [24]. Further studies are required to ascertain the oncogenic risk of mastauxe.

Neonatal mastauxe requires simple observation and parental reassurance. Repeated expression of witch’s milk by manual squeezing has been strongly discouraged as it is believed to prolong milk secretion and introduce infection [25]. Buehring [26] while studying the diagnostic utility of witch’s milk in 106 infants, confirmed the former and refuted the later. Repeated manual emptying of glands, indeed, prolonged milk secretion as long as 24 weeks and significantly increased the amount of milk from 20 µl to 1500 µl per sample. Despite repeated handling of breast, none of the 106 infants developed infective complications such as mastitis or abscess. Finally, Athena is amused by Buehring’s speculation that witch’s milk analysis could be a useful adjunct in the diagnosis of certain inborn errors of metabolism. Concentration of phenylalanine in witch’s milk is higher than that in neonatal blood. Therefore, hypothetically, witch’s milk analysis may increase the diagnostic yield of neonatal screening for phenylketonuria [26].

## Footnotes

**Source of Support:** Nil

**Conflict of Interest:** The author is Editor of the journal. But he did not take part in the evaluation or decision making of this manuscript. The manuscript has been independently handled by two other editors.

